# MFGE8, ALB, APOB, APOE, SAA1, A2M, and C3 as Novel Biomarkers for Stress Cardiomyopathy

**DOI:** 10.1155/2020/1615826

**Published:** 2020-06-24

**Authors:** Xiao-Yu Pan, Zai-Wei Zhang

**Affiliations:** ^1^Department of Clinical Medical College, Jining Medical University, Jining, Shandong 272067, China; ^2^Department of Cardiology, Jining No. 1 People's Hospital, Jining, Shandong 272011, China; ^3^Cardiovascular Research Institute, Jining No.1 People's Hospital, Jining, Shandong 272011, China

## Abstract

**Background:**

Stress cardiomyopathy (SCM) is a transient reversible left ventricular dysfunction that more often occurs in women. Symptoms of SCM patients are similar to those of acute coronary syndrome (ACS), but little is known about biomarkers. The goals of this study were to identify the potentially crucial genes and pathways associated with SCM.

**Methods:**

We analyzed microarray datasets GSE95368 derived from the Gene Expression Omnibus (GEO) database. Firstly, identify the differentially expressed genes (DEGs) between SCM patients in normal patients. Then, the DEGs were used for Gene Ontology (GO) and Kyoto Encyclopedia of Genes and Genomes (KEGG) pathway enrichment analysis. Finally, the protein-protein interaction (PPI) network was constructed and Cytoscape was used to find the key genes.

**Results:**

In total, 25 DEGs were identified, including 10 upregulated genes and 15 downregulated genes. These DEGs were mainly enriched in ECM-receptor interaction, dilated cardiomyopathy (DCM), human papillomavirus infection, and focal adhesion, whereas in GO function classification, they were mainly enriched in the extracellular region, positive regulation of the multicellular organismal process, establishment of localization, and intracellular vesicle.

**Conclusion:**

Seven hub genes contained APOE, MFGE8, ALB, APOB, SAA1, A2M, and C3 identified as hub genes of SCM, which might be used as diagnostic biomarkers or molecular targets for the treatment of SCM.

## 1. Introduction

Stress cardiomyopathy, also known as takotsubo cardiomyopathy (TTC), was first reported in Japan in 1990 [1], characterized by transient systolic and diastolic left ventricular (LV) dysfunction with multiple wall dyskinesia, most commonly occurring in postmenopausal women, especially in populations with a recent history of mental or physical stress [2]. The reversibility of cardiac insufficiency is one of the most prominent features of the disease. It is sometimes considered fairly benign, but it has a 4.1% in-hospital mortality rate, especially in the early stages analogous to acute coronary syndrome (ACS), and is also likely to have cardiogenic shock and fatal arrhythmias [3]. It estimates that 1 to 2% of suspected ACS patients were eventually diagnosed with stress cardiomyopathy.

The exact pathophysiology of stress cardiomyopathy is unknown and seems to be associated with excess plasma catecholamine, which is caused by stress conditions. Myocardial ischemia appears to play a crucial role in takotsubo syndrome, both in human heart muscle specimens and in experimental models of SCMP. Most cases occur in patients with risk factors for endothelial dysfunction [4].

Most patients with stress-induced cardiomyopathy (>95%) have electrocardiogram abnormalities that typically show ischemic ST-segment and T-wave changes, but his appearance is most likely to associate the presence of ACS than SCM [5, 6]. Previous studies have shown that cardiac biomarkers such as cardiac troponin T or I, creatine-kinase myocardial band (CK-MB), and b-type natriuretic peptide (BNP) may play a role in the early clinical recognition of SCM, but the accuracy is low [7, 8]. In patients with suspected stress cardiomyopathy, transthoracic echocardiography with color and tissue doppler is the preferred noninvasive imaging trial [5]. A recent study reports that STE echocardiographic index may be more accurate and unique than traditional echocardiography for the early detection of subtle abnormalities [9]. Because of the reversibility of SCM's condition, it is not possible to determine the diagnosis at the time of presentation, although some clinical features are highly predictive of stressful myocardial disease [10].

In this study, we further revealed biomarkers related to SCM by analyzing gene expression profile (GSE95368) deposited by Yvonne Edwards et al. (2017). Identifying key genes and pathways contributes to a better understanding of the pathophysiological mechanism of disease development, which provides innovative ideas for the diagnosis and treatment of SCM.

## 2. Materials and Methods

### 2.1. Data Sources

Gene expression data of GSE95368 is available for download from the NCBI Gene Expression Omnibus (GEO; http://www.ncbi.nlm.nih.gov/geo). The expression profiling arrays were generated using the GPL23119 platform (SOMAscan human proteomic 1.3k assay). A total of 21 serum samples in this database, including 15 from SCM patients and 6 from normal serum samples, were analyzed.

### 2.2. Data Preprocessing and Identification of DEGs

Genetic matrix files and platform files were downloaded to eliminate errors and make the experimental group comparable between control groups, so the obtained data were standardized. The estimation package is based on the KNN (k-nearest neighbor) algorithm is used to fill the missing values. After this, the probes were converted into gene symbols on the basis of the annotation platform file. If there are multiple probes corresponding to a gene, take the average as the final value. If the probe without gene symbol was removed, the normalized between array function in the limma package is applied to standardize the data. Then, the expression data were log2 transformed and the limma functional package in R software was used to compare gene expression in SCM and control samples to identify DEGs. The screening criteria for DEGs were *p* value < 0.05 and ∣log2foldchange (FC) | ≥1.

### 2.3. GO and KEGG Pathway Enrichment Analysis of DEGs

Database for Annotation, Visualization and Integrated Discovery (DAVID) (https://david.ncifcrf.gov) and KOBAS (http://kobas.cbi.pku.edu.cn) were applied for GO annotation and KEGG pathway analysis. Because the KOBAS online tool uses gene ID for data analysis, so we first use DAVID to convert the gene symbol of DEGs into gene ID and then use KOBAS for GO and KEGG enrichment analysis of DEGs. Finally, we use R software to visualize the results, to further understand the potential functions of the identified DEGs.

### 2.4. PPI Network Analyses

PPI network analyses can show the functional link between proteins and proteins, using string software (http://www.string-db.org) for PPI network analysis of differential genes. When building a protein interaction network, the settings are all set by default. Subsequently, Cytoscape software was applied to visualize and analyze the PPI network.

### 2.5. Module Analysis and Selection of Hub Genes

Molecular Complex Detection (MCODE) can find the interacting dense region in the PPI network, and the dense regions of interest can also be extracted and visualized. So we use MCODE to discover modules across the network. The hub genes were identified by using the plug-in cytoHubba of the Cytoscape software, including Maximal Clique Centrality (MCC), Density of Maximum Neighborhood Component (DMNC), and Maximum Neighborhood Component (MNC).

## 3. Results

### 3.1. Identification of DEGs

**Figure 1:**
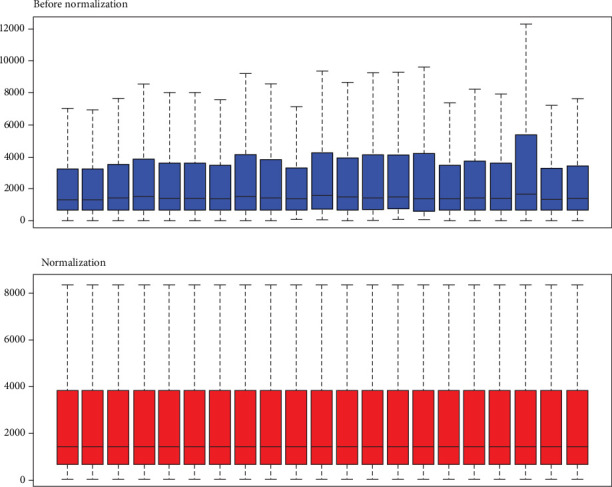
Standardization of gene expression. The blue bar represents the data before normalization, and the red bar represents the normalized data.

**Figure 2:**
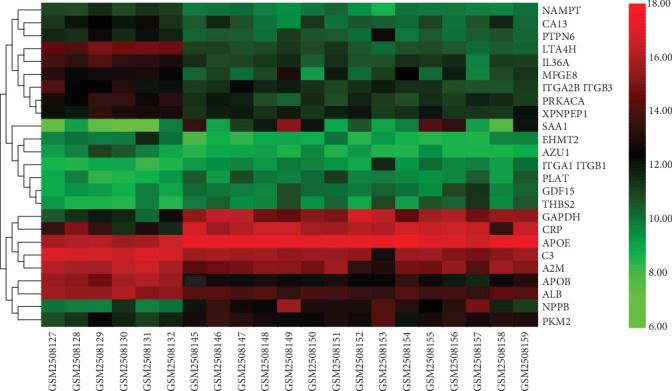
Heatmap results of DEGs. Abbreviation: DEGs, differentially expressed genes.

**Figure 3:**
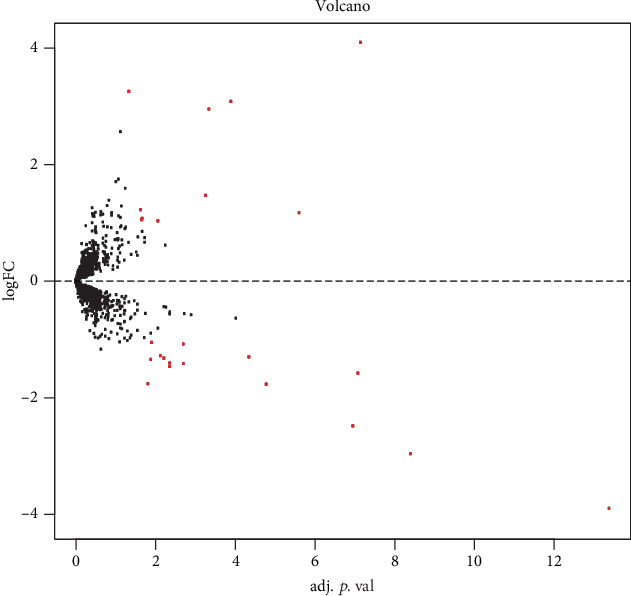
Differential expression of data between two sets of samples.

The results of standardizing the matrix file are shown in Figure 1. We identified a total of 25 DEGs in SCM samples compared with normal samples, including 10 upregulated genes and 15 downregulated genes, which were statistically significant (adjusted *p* < 0.05, ∣log fold change (FC) | >1) (Table 1). The cluster heatmap plot and volcano plot of the DEGs are shown in Figures 2 and 3.

### 3.2. GO Enrichment Analysis of DEGs

**Figure 4:**
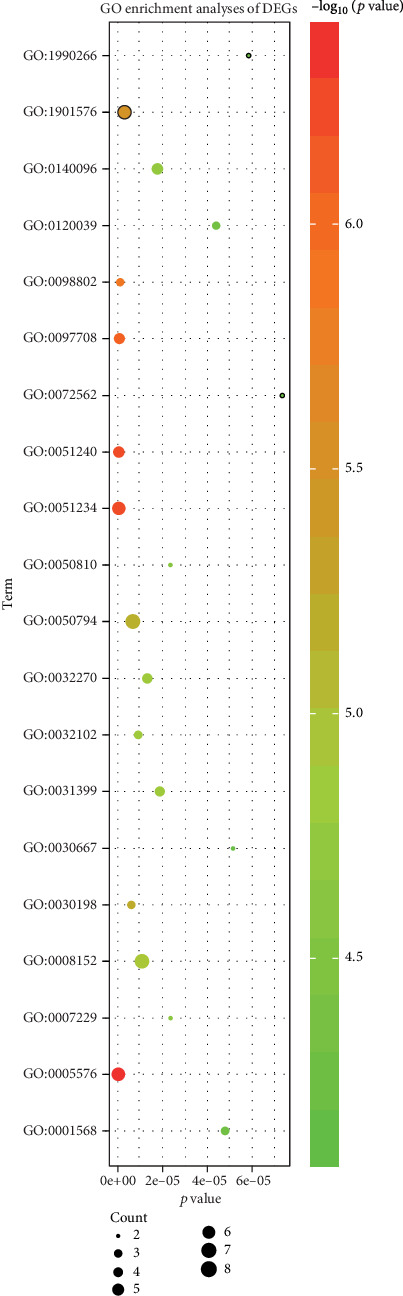
The DEGs significantly enriched GO (top 20). Abbreviation: DEGs, differentially expressed genes; GO, Gene Ontology.

The GO analysis consists of biological processes (BP), cellular component (CC), and molecular function (MF) terms. The different genes with adjusted *p* value < 0.05 were obtained from GO functional enrichment. The GO enrichment analysis results reveal that the DEGs were mainly involved in the extracellular region, positive regulation of the multicellular organismal process, establishment of localization, and intracellular vesicle (Table 2, Figure 4).

### 3.3. KEGG Pathway Analysis of DEGs

**Figure 5:**
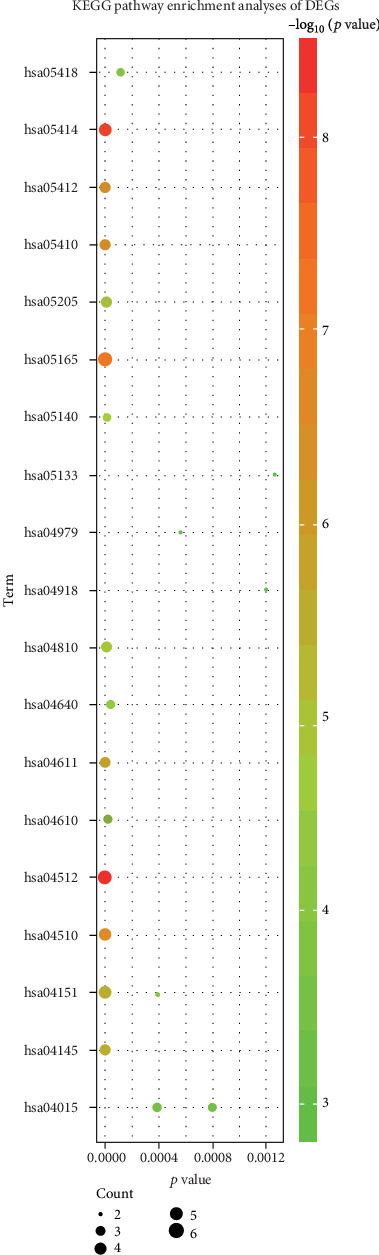
KEGG pathway analysis of DEGs. Abbreviation: KEGG: Kyoto Encyclopedia of Genes and Genomes; DEGs: differentially expressed genes.

The KEGG pathways of the DEGs were analyzed using DAVID and KOBAS. The top 20 of the KEGG pathways is shown in Table 3 and Figure 5, the DEGs chiefly enriched in ECM-receptor interaction, dilated cardiomyopathy (DCM), human papillomavirus infection, and focal adhesion.

### 3.4. Establishing the PPI Network, Conducting Module Analysis, and Selection of Hub Genes

**Figure 6:**
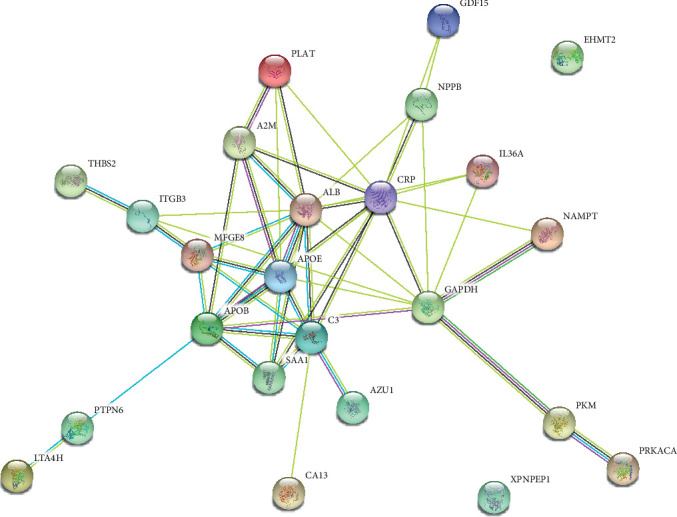
Results of PPI network analysis of DEGs. Abbreviation: PPI: protein-protein interaction; DEGs: differentially expressed genes.

**Figure 7:**
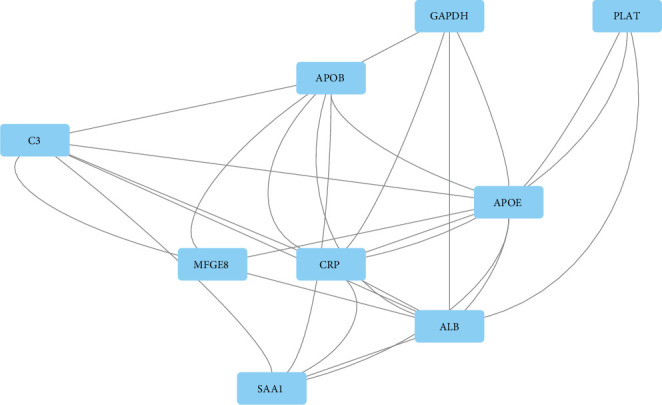
PPI network of module. Abbreviation: PPI: protein-protein interaction.

**Figure 8:**
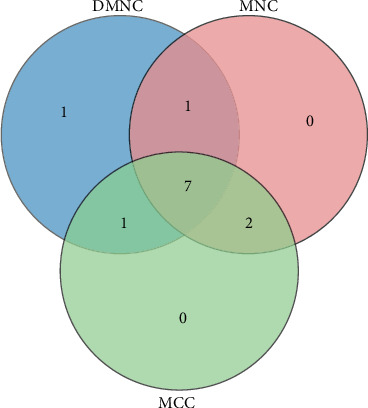
Venn diagram of common hub genes based on three methods. Abbreviation: MCC: Maximal Clique Centrality; DMNC: Density of Maximum Neighborhood Component; MNC: Maximum Neighborhood Component.

Use the string online tool to create a PPI network to gain a better understanding of the biological properties of DEGs. There were 24 nodes and 51 edges in this network, as shown in Figure 6. Subsequently, Cytoscape is applied to confirm a vital module throughout the network; the most significant modules were selected, as shown in Figure 7. Nine key genes were identified, including SAA1, C3, CRP, ALB, APOE, APOB, MFGE8, GAPDH, and PLAT, finally utilizing the cytoHubba plug-in to determine APOE, MFGE8, ALB, APOB, SAA1, A2M, and C3 as the hub gene, as shown in Figure 8 and Table 4.

## 4. Discussion

In this study, we performed an integrated analysis of gene expression profiles from serum samples without/with SCM aiming to identify the DEGs, related key signaling pathways, and hub genes for the disease. A total of 25 DEGs, including 10 upregulated and 15 downregulated genes, were identified from the GSE95368 database. The GO enrichment analysis showed that these differential genes associated with SCM were mainly enriched in the extracellular region, positive regulation of the multicellular organismal process, establishment of localization, and intracellular vesicle. From the KEGG pathway enrichment analysis, we identified that these DEGs were mainly enriched in the pathway of the ECM-receptor interaction and dilated cardiomyopathy (DCM). Through the construction and module analysis of the PPI network, we identified 9 key genes, including SAA1, C3, CRP, ALB, APOE, APOB, MFGE8, GAPDH, and PLAT. Finally, APOE, MFGE8, ALB, APOB, SAA1, A2M, and C3 are regarded as hub genes for the development of SCM.

MFGE8, a secreted glycoprotein, is associated with a variety of pathophysiological processes, including anti-inflammatory [11], antifibrosis [12], antiatherosclerosis [13], and inhibition of cardiac hypertrophy [14]. Recent studies have shown that MFGE8 is a part of the arterial inflammatory signaling network that promotes endothelial cell apoptosis [15]. MFGE8 is an inflammatory mediator that coordinates multiple cellular interactions and is involved in the pathogenesis of various diseases. In a study investigating postischemic injury, MFGE8 may alleviate postischemic injury by integrin beta3-dependent inhibition of inflammatory bodies [15]. Cardiomyocyte ischemia plays a critical role in the development of SCM, so we propose that MFGE8 may play an important role in SCM by participating in the process of myocardial ischemia.

C3 is the most abundant complement component in serum, mainly macrophage and liver synthesis, and plays an important role in complementing the classical activation pathway and bypass activation pathway [16]. C3 increases are common in the early stages of some acute inflammation or infectious diseases [17]. Although the specific mechanism of SCM is unclear, most patients are subjected to stress status. C3 is one of many neurohumoral factors released as a result of a series of adaptive responses in the body under stress [18]. So C3 may be involved in the pathological process of SCM through this pathway. SAA promotes chemotaxis of monocytes and neutrophils and plays a key role in various functions such as lipoprotein metabolism, cholesterol transport, and host defense [19]. Previous studies have shown that SAA1 plasma levels have increased dramatically in response to tissue damage, infection, and various emergencies [20, 21]. So SAA1 is also involved in the pathogenesis of SCM, and its mechanism is similar to that of C3.

Alpha-2-macroglobulin (A2M) is a broad-spectrum protease-binding protein of the vertebrate innate immune system that prevents pathogen invasion [22, 23]. In our study, both A2M and C3 were enriched in the complement and coagulation cascade pathway, suggesting an important role in coagulation. A recent study suggests that SCM is a prethrombotic state [24], which is consistent with our findings, so we speculate that A2M and C3 may be involved in the SCM pathological process through this pathway.

Serum albumin (ALB) is synthesized in the liver and is the most abundant protein in vertebrate plasma. Its main function is to maintain plasma colloid osmotic pressure and participate in the transport of various substances [25, 26]. We found that ALB is mainly associated with the synthesis of thyroid hormones, which increase in response to human mood swings and emergencies [27]. So thyroid hormone is involved in the process of SCM, and therefore, ALB is also likely to be involved in the pathogenesis of SCM. In addition, several studies have shown that serum ALB has a predictive value for a variety of thrombotic diseases [28, 29]. As mentioned above, SCM is also a prethrombotic state, so we guess that serum ALB also seems to have some predictive effect on SCM.

Apolipoprotein is a protein that can bind and transport blood lipids to tissues for metabolism and utilization [30]. A large number of studies have found that apolipoprotein gene mutation can affect blood lipid metabolism and utilization, thus growing up to be a risk factor for cardiovascular and cerebrovascular diseases, diabetes, and other diseases [31, 32]. APOE and APOB are members of the apolipoprotein family, and so far, no studies have shown a direct relationship between apolipoprotein and SCM. Previous studies have shown that diabetes is a risk factor for SCM, and hyperlipidemia is a risk factor for diabetes [33, 34], so it can be speculated that hyperlipidemia is a potential risk factor for SCM, so compared with the normal population, APOE and APOB genes are upregulated in SCM patients.

In this experiment, we analyzed gene chips to obtain SCM possible key genes and related pathway information. However, due to the defects of the study itself, the conclusion needs basic and clinical experimental verification. Most regrettably, due to the limited experimental conditions, the conclusions drawn in this paper cannot be further investigated. But we hope to be able to provide new ideas for SCM diagnosis based on this study and expect other scientific researchers to further explore this.

## 5. Conclusions

In summary, our study provides an integrated bioinformatics analysis of DEGs of SCM. In the present study, we identified some key genes and pathways. However, the key genes and signaling pathways related to SCM derived from this study still need further experimental verification due to the defects of analytical methods and sample size.

## Figures and Tables

**Table 1:** Screening upregulated and downregulated DEGs.

DEGs	Gene symbol
Upregulated (15)	LTA4H APOB ALB IL36A PRKACA NAMPT XPNPEP1 ITGA2B ITGB3 CA13 C3 EHMT2 PTPN6 AZU1 A2M MFGE8
Downregulated (10)	GAPDH APOE CRP NPPB PKM2 GDF15 PLAT ITGA1 ITGB1 THBS2 SAA1

Abbreviation: DEGs: differentially expressed genes.

**Table 2:** GO enrichment analysis of differentially expressed genes.

Term	Description	Count	*p* value
GO:0005576	Extracellular region	7	4.42*E*-07
GO:0051240	Positive regulation of the multicellular organismal process	5	5.94*E*-07
GO:0051234	Establishment of localization	7	6.12*E*-07
GO:0097708	Intracellular vesicle	5	8.56*E*-07
GO:0098802	Plasma membrane signaling receptor complex	3	1.31*E*-06
GO:1901576	Organic substance biosynthetic process	7	3.12*E*-06
GO:0030198	Extracellular matrix organization	3	6.13*E*-06
GO:0050794	Regulation of cellular process	8	6.88*E*-06
GO:0032102	Negative regulation of response to external stimulus	3	9.39*E*-06
GO:0008152	Metabolic process	8	1.10*E*-05
GO:0032270	Positive regulation of cellular protein metabolic process	4	1.32*E*-05
GO:0140096	Catalytic activity, acting on a protein	5	1.78*E*-05
GO:0031399	Regulation of protein modification process	4	1.89*E*-05
GO:0007229	Integrin-mediated signaling pathway	2	2.37*E*-05
GO:0050810	Regulation of steroid biosynthetic process	2	2.37*E*-05
GO:0120039	Plasma membrane-bounded cell projection morphogenesis	3	4.40*E*-05
GO:0001568	Blood vessel development	3	4.80*E*-05
GO:0030667	Secretory granule membrane	2	5.16*E*-05
GO:1990266	Neutrophil migration	2	5.84*E*-05
GO:0072562	Blood microparticle	2	7.34*E*-05

Abbreviation: GO: Gene Ontology.

**Table 3:** KEGG pathway analysis of DEGs.

Pathway	ID	Count	*p* value	Genes
ECM-receptor interaction	hsa04512	5	4.03*E*-09	ITGA1|ITGB3|THBS2|ITGB1|ITGA2B
Dilated cardiomyopathy (DCM)	hsa05414	5	6.84*E*-09	ITGA1|ITGB3|PRKACA|ITGB1|ITGA2B
Human papillomavirus infection	hsa05165	6	8.06*E*-08	THBS2|ITGB1|ITGB3|PRKACA|ITGA1|ITGA2B
Focal adhesion	hsa04510	5	2.31*E*-07	ITGA1|ITGB3|THBS2|ITGB1|ITGA2B
Arrhythmogenic right ventricular cardiomyopathy (ARVC)	hsa05412	4	2.55*E*-07	ITGA1|ITGB3|ITGB1|ITGA2B
Hypertrophic cardiomyopathy (HCM)	hsa05410	4	4.65*E*-07	ITGA1|ITGB3|ITGB1|ITGA2B
Platelet activation	hsa04611	4	1.60*E*-06	ITGB3|PRKACA|ITGB1|ITGA2B
Phagosome	hsa04145	4	3.52*E*-06	ITGB3|THBS2|ITGB1|C3
PI3K-Akt signaling pathway	hsa04151	5	3.71*E*-06	ITGA1|ITGB3|THBS2|ITGB1|ITGA2B
Proteoglycans in cancer	hsa05205	4	1.08*E*-05	ITGB3|PRKACA|ITGB1|PTPN6
Regulation of actin cytoskeleton	hsa04810	4	1.32*E*-05	ITGA1|ITGB3|ITGB1|ITGA2B
Leishmaniasis	hsa05140	3	1.89*E*-05	ITGB1|PTPN6|C3
Complement and coagulation cascades	hsa04610	3	2.29*E*-05	PLAT|A2M |C3
Hematopoietic cell lineage	hsa04640	3	4.15*E*-05	ITGA1|ITGB3|ITGA2B
Fluid shear stress and atherosclerosis	hsa05418	3	0.000117572	ITGB3|PLAT|ITGA2B
Cholesterol metabolism	hsa04979	2	0.00056172	APOE|APOB
Thyroid hormone synthesis	hsa04918	2	0.001195438	PRKACA|ALB
Pertussis	hsa05133	2	0.001258577	ITGB1|C3
Rap1 signaling pathway	hsa04015	3	0.00038722786	ITGB3|ITGB1|ITGA2B
ECM-receptor interaction	hsa04512	5	4.03*E*-09	ITGA1|ITGB3|THBS2|ITGB1|ITGA2B

Abbreviation: KEGG: Kyoto Encyclopedia of Genes and Genomes; DEGs: differentially expressed genes.

**Table 4:** Hub genes based on cytoHubba.

Projects	Methods in cytoHubba		
	MCC	MNC	DMNC
Gene symbol top 10	**ALB**	**ALB**	**SAA1**
	**APOE**	CRP	**C3**
	CRP	**APOE**	**A2M**
	**APOB**	**APOB**	PLAT
	**C3**	GAPDH	**APOB**
	**SAA1**	**C3**	**APOE**
	**A2M**	**A2M**	IL36A
	GAPDH	**MFGE8**	**MFGE8**
	**MFGE8**	**SAA1**	**ALB**
	PLAT	NPPB	NPPB

Bold gene symbols were the overlap hub gene. Abbreviation: MCC: Maximal Clique Centrality; DMNC: Density of Maximum Neighborhood Component; MNC: Maximum Neighborhood Component.

## Data Availability

The data used to support the findings of this study are included within the supplementary information file.

## Supplementary Materials

Supplementary Materials GSE95368 datasets were downloaded from GEO (http://www.ncbi.nlm.nih.gov/geo/).
